# Commentary

**DOI:** 10.1093/schbul/sbae191

**Published:** 2025-02-21

**Authors:** Richard S E Keefe

**Affiliations:** Department of Psychiatry, Duke University Medical Center, Durham, NC 27710, United States

The position paper by Horan et al^1^ raises a series of issues regarding the current conduct of clinical trials for the treatment of cognitive impairment in schizophrenia, which has become known as CIAS. Notably, no authors from any regulatory agency were included. I was told that Food and Drug Administration (FDA) representatives are planning on their own publication to address these issues. We eagerly await their viewpoint. The Measurement and Treatment Research to Improve Cognition in Schizophrenia (MATRICS) process stimulated drug companies to invest in CIAS programs. If companies have retreated from this important area of work due to challenges with regulatory requirements, then it is urgent that FDA and others respond publicly. However, if the reduced investment over time is due to confusion about why CIAS programs have failed, then this and other forums are essential to clarify what aspects of this work are challenging and how those challenges can be addressed.

Since the MATRICS process began in 2000, 50 molecules have been studied in phase 1 trials registered on clinicaltrials.gov that were initiated in a CIAS development program. Of those, 39 progressed to phase 2 and two to phase 3.^2^ Efficacy of a drug is almost never evident before phase 2, so to be clear, the issue in CIAS drug development is not “replication failure.” Although definitions of what is a positive result may differ, fewer than 5 compounds have demonstrated efficacy. Two of these were progressed to phase 3. One of those programs, the alpha7 nicotinic agonist encenicline, was negative, consistent with the 8 other failed programs investigating this mechanism. The other program, investigating the glycine transporter-1 inhibitor iclepertin, is nearing the end of three phase 3 trials. Given that there have only been two phase 3 programs in CIAS, and one of them is currently underway, it is premature to make any conclusions about whether the phase 3 process is effective. Therefore, it may be more constructive for discussion about the sensitivity of CIAS clinical trial methods to be limited to phase 2 trials.

Drug companies and researchers are generally free to follow whatever methods they believe will address the efficacy of their compounds in phase 2 trials, so at first glance, it would seem odd that a publication authored primarily by drug company employees would be devoted to the challenges presented by regulatory agencies based upon the results of negative phase 2 trials. It is logical to ask why drug company research teams have not simply chosen the methods that they see as best, and then if they have positive results, petition the FDA as to the benefits of their improved methods. Unfortunately, because of the high cost of phase 3 trials, industry researchers desire a high degree of certainty that their phase 3 methodology will pass muster with regulatory agencies before they begin, especially since phase 3 methods are often developed before phase 2 results are completed. Drug companies upper management, stockholders, and other investors thus have difficulty stomaching the financial risk of changing methodologies from earlier to later phases. These concerns are natural and often out of the hands of researchers designing trials, although the related discussions can border on superstition, such as the surprisingly common question as to whether a drug’s benefit depends upon which verbal memory word list or version of symbol coding was used in earlier phase trials. It is rare in these discussions to hear someone voice the concern that a drug with such a fragile treatment effect might not be sufficient to improve patients’ lives.

Horan et al raise important issues about the implementation of the endpoints in CIAS trials, particularly the MATRICS Consensus Cognitive Battery (MCCB). To provide some relevant personal background on my perspective, I have been involved directly or indirectly in the data collection process for many of the phase 2 and all phase 3 trials in CIAS, including overseeing training for over 10 000 raters and data review for close to 100 000 MCCB assessments. From that experience and the data we have collected along the way, I wanted to provide support for some of the viewpoints they assert and point out a few misconceptions based on the data that we have collected over the years. While readers may sympathize with the frustrations of those devoting careers to finding treatments for CIAS only to be repeatedly thwarted by negative trials, the path forward should be evidence-based.

## Is the MCCB Too Long?

First, it is important to acknowledge that the psychometric properties of the MCCB, which are essential to detect treatment effects, are excellent. The cognitive composite score generated from the MCCB, which is almost always the primary endpoint for CIAS trials, has excellent reliability and is highly sensitive to differences among individuals.^3^ I have never heard a reputable schizophrenia cognition expert say that the MCCB composite score would not be sensitive to a true treatment effect in people with schizophrenia. However, the MCCB includes 10 tests and requires approximately 90 minutes to complete. The duration of the battery has been viewed by some as being burdensome to patients and the resources of sites conducting trials. With regard to patients, my colleagues and my perception from training sites and reviewing rater data is that patient complaints about the assessment have been rare, and the rate of refusal has been consistent with other clinical assessments. As evidence, the overall missing data rate at the first visit is less than 0.3%.^3^ The MCCB composite score is considered valid if data are non-missing on 4 or more of the 7 cognitive domains. In our published analysis of an antipsychotic trial using the MCCB, a missing MCCB composite score occurred only once in 646 assessments.^4^

So it is clear that the MCCB is not so burdensome that missing data becomes a problem. But could the MCCB be less demanding? Certainly. As Horan et al note, even a very small number of cognitive tests can generate similar test-retest reliability and correlations with functioning as found with the MCCB.^5–9^ However, the rationale for the requirement of a standard set of 7 domains was not for the purpose of identifying specific drug effects as much as it was to prohibit bias. Its intention was to prevent drug companies from stacking the deck in favor of their compound by only including domains or tests that are expected to improve with treatment while excluding tests that may worsen. Given that some cognitively active drugs can facilitate some aspects of cognition while impairing others (eg, stimulants like methylphenidate and caffeine can simultaneously improve attention while they increase impulsive errors of commission^10^) and that FDA’s primary concern is patient safety, the requirement is not unreasonable. However, Horan et al make the excellent point that a general cognitive deficit accounts for almost all of the impairment in CIAS, so the likelihood that a drug will have specific effects is small. Although not required by regulators, given that the current standard for CIAS trials is to assess cognition several times over the course of the 2-6 months of the trial, perhaps regulators could compromise by accepting a strategy of using a briefer efficacy endpoint assessment of the general cognitive impairment that is assessed regularly, accompanied by a more extensive “safety” assessment of all 7 domains at baseline and end of study. This proposal would allow less cost and patient burden, while also enabling regulators to check for cognitive worsening in relevant cognitive domains.

## Is Training Personnel to Conduct the MCCB Too Burdensome?

Many site Principal Investigators complain about the duration of the assessment and the training process for their staff members. Our experience with training was that personnel were frequently being asked to conduct an assessment for which they had little background, and the pathway to competence was steep. This challenge was significantly exacerbated as a result of the coronavirus disease 2019 pandemic and the changes in workplace commitment that followed. International trials have created additional burdens for training, translation, and data review of the MCCB. However, objections from highly trained clinician-scientists about the requirement of training for a technical skill are perplexing. Clinical trials in many other Central Nervous System (CNS) disorders require personnel with advanced degrees as well as successful completion of instrument-specific training programs, such as the Clinician-Administered PTSD Scale for DSM-5 (CAPS-5). In Alzheimer’s disease trials, advanced technical degrees are required to administer Magnetic Resonance Imaging (MRI) and Positron Emission Tomography (PET) measures, in addition to protocol-specific training. No one seems to balk at this process. The challenge in CIAS trials may be that advanced degrees are not required to collect MCCB data, and so the personnel who are identified by site investigators to fill that role do not have significant experience. I have interacted with many testers who have arrived at their certification sessions to declare that they were informed about their role as cognitive tester so recently that they had no time to prepare. I have also had experiences with many testers without psychology backgrounds who refuse to follow basic test instructions, completely oblivious to the rationale behind concepts such as test standardization. Is this a problem with the test or with the lack of structure that has been put in place to support these important studies? Perhaps this problem arises because, from a distance, cognitive testing like the MCCB appears to be easy. I am not aware of any Alzheimer’s disease researchers calling for reduced training procedures for MRI and PET tech personnel in their trials, probably because they are already a part of the institutional infrastructure and do not add costs.

## Why are Newer Technologies for Cognitive and Functional Assessment Not Accepted and Utilized?

Technology has created the opportunity for digital endpoints with equal or better reliability than the paper-and-pencil methods that were standard when the MCCB battery was constructed 25 years ago. However, acceptance of these endpoints requires extensive validation with data collected from large databases and flexible regulatory processes that encourage innovation. While some test developers have taken steps toward validation, others remain in aspirational stages. Regulatory agencies seem to be well-intentioned to encourage innovation, but their implementation has fallen short. The FDA Clinical Outcome Assessment Qualification website (https://www.fda.gov/drugs/clinical-outcome-assessment-coa-qualification-program/clinical-outcome-assessments-coa-qualification-program-submissions) lists that it has accepted 66 applications into the program. Many of these have languished for over a decade, while only 5 have been qualified. All 5 were patient-reported outcomes, which are far less complex than performance-based measures like cognitive assessments. It is not an efficient use of the citizenry for some government agencies to encourage speedy innovation while others block traffic. Government agencies need to work together better to encourage and support technology development that will increase the speed and efficiency of CIAS trials. Drug companies have also done little to foster innovation in CIAS. Most companies are structured in such a way that funding for innovative assessments in therapeutic areas without a product is sparse since there are no revenue streams to draw from. Public-private partnerships, with the involvement of key regulators, can be developed to advance the field further and ease the burden of CIAS trials. Societies like the International Society for CNS and Clinical Trials Methodology (ISCTM) would be very open to such collaborative advances.

## How Much Benefit is Meaningful?

Although not raised directly by Horan et al, an additional challenge to drug development in CIAS is the absence of any consensus on the definition of a “clinically meaningful effect,” which is of great importance to the FDA and other regulatory agencies. The cold fact is that we really do not know. Any choice is based upon theoretical notions that are yet unsupported with data. So, how do we determine a cutoff for what amount of change is sufficient to warrant the conclusion that a patient’s cognition has changed for the better? It is incumbent upon a company applying for a drug’s approval to demonstrate that it creates this meaningful effect, even if no such definition exists. The biggest problem with the definition of clinically meaningful effect in CIAS is that it requires dichotomization—yes/no to whether the drug worked enough to matter—on measures that are continuous by nature, which reduces information in any analysis.^11^ Further, such cutoffs need to be pre-specified and are always arbitrary. How this dumbing-down of the statistical methods to understanding efficacy has survived among regulators is perplexing. Not only does the approach violate statistical understanding, but it requires a value judgment about how much change matters to a broad swath of the underserved population with schizophrenia, from those who are desperate to keep a good job to those who are unable to figure out how to live independently. The initial perspective from the FDA at the MATRICS meetings and publications was that the meaning of a small but statistically significant improvement could not be determined, so a co-primary measure of functioning or functional capacity would be needed for CIAS trials. Why should drug companies be forced to identify a clinically meaningful effect size while at the same time showing statistically significant benefit on a measure of functional capacity? This hurdle seems unnecessarily high and redundant. Alzheimer’s researchers have proposed the use of a single cognition-functioning composite score that would streamline the assessment of efficacy in their trials, and regulators have been open to this strategy. An analogous approach of cognitive performance tools and either functional measures or interview-based assessment in CIAS trials should be considered.

Two timely events in the treatment of schizophrenia are relevant to this discussion. First, the results of the phase 3 Boehringer Iclepertin study should be forthcoming in the early part of 2025. This program directly followed almost all of the MATRICS recommendations, including the use of the MCCB composite score as the primary endpoint, which demonstrated efficacy in their phase 2 trial.^12^ If the phase 3 trials are positive, how will we view the MATRICS recommendations and the MCCB then? Will they be forever accepted as gold standard methods? If negative, does that mean that all of the methods should be abandoned? Hopefully, these discussions will be governed not by superstition and the exaggerated power of precedent, but by thoughtful scientific discourse.

The other major event in schizophrenia treatment was the recent FDA approval of Xanomeline, the first approval of an antipsychotic with a mechanism of action that does not directly block dopamine receptors or cause dopamine-blocking side effects. It will likely change the landscape of drug development for schizophrenia. Several previous drug development programs targeted non-dopamine-blocking strategies for psychosis but were not successful. Luckily for our patients, the team of investigators researching xanomeline—and their investors—persisted despite the small odds of success that faced them. Part of the lesson of Xanomeline’s success is that to provide further help to people with schizophrenia, we need to keep generating opportunities. We need more shots on goal.

But why has schizophrenia research been so limited in the number of shots on goal to find novel treatments for what is one of the most devastating illnesses to face humanity? One of the concerns expressed by Horan et al regarding drug development for CIAS was the cost of these programs. Here, it is helpful to compare CIAS to other disease areas, where investments are orders of magnitude greater than for CIAS.^13^ As an example within CNS, phase 3 trials for CIAS have cost far less than the average phase 3 trial for Alzheimer’s disease, which requires a longer duration and expensive assessments such as MRI and PET. In fact, there were a whopping 878 phase 3 trials in Alzheimer’s disease from 1995 to 2021, costing over $24 billion,^14^ dwarfing the magnitude of the drug development effort for cognition in schizophrenia. And while 5 of the programs during that period (and two since 2022, lecanemab and donanemab) have been successful in identifying drugs that can reduce the progressive cognitive decline in Alzheimer’s disease in phase 3, the cognitive benefit has been small, with the largest of these *d* = 0.36. It is noteworthy that the effect sizes for lecanemab and donanemab were slightly higher than for previous drugs, possibly suggesting improvements in our understanding of Alzheimer’s disease clinical trials methodology and the disease process itself.

The decades-long public investment in Alzheimer’s disease research has generated science that has not only helped us home in on more accurate targets for preventing cognitive decline but has solved some of the aspects of clinical trials methods that continue to vex CIAS clinical trials, such as the identification of the appropriate patients with refined biomarkers. In addition, several highly functioning networks of clinical trial sites for Alzheimer’s disease in the United States have been extensively supported by funding from various sources, including industry/foundation collaborations, the Veterans Health Administration (VA), and National Institute of Aging (NIA), directly resulting in an ongoing healthy collaboration between drug companies and academic medical centers. There are similar numbers of networks in Europe and Asia. As a result of the absence of government and industry investment in similar networks for schizophrenia, fewer academic research centers support schizophrenia clinical trials, and even fewer are involved in schizophrenia cognition trials. While the National Institue of Mental Health (NIMH) Accelerated Medicines Program in schizophrenia holds promise for helping to establish productive research networks, especially in high-risk conditions, an investment in clinical trials sites from NIMH, private foundations, and especially drug companies that stand to benefit financially from innovative strategies and methods in CIAS trials, is sorely needed and overdue. Many of the concerns raised by Horan et al could be addressed by the establishment of existing site networks with certified personnel for screening, testing, and assessing patients for CIAS trials.

In the United States, the number of people with Alzheimer’s disease is about twice as large as the number of people living with schizophrenia. Yet, in 2024, the National Institutes of Health invested more than 8 times as much in research for Alzheimer’s disease than in schizophrenia.^15,16^ How is it possible that a staggering amount of industry and government resources are invested in Alzheimer’s disease to a unified choir of encouraging voices from academics, government leaders, and industry executives, yet the investment from the same resources in schizophrenia is paltry? It is no wonder that industry researchers who have invested their careers in CIAS work want an easier path forward. Yet, while refining our methods to make the regulatory pathway less burdensome may help, we should also be demanding far more as constituents of our society and our governments. It seems illustrative that the tagline of the latest ad campaign to fight Alzheimer’s is “We all know someone with Alzheimer’s.” For schizophrenia, it seems as though an apt tagline would be “We all ignore someone with schizophrenia.” It is grotesquely unjust that resources are directed more to those who have had decades of normal life than to those who have decades of suffering ahead of them. While some of the proposed changes to the methods of CIAS trials may expedite treatments to reduce schizophrenia patients’ suffering, we should not ignore that a far heavier burden is the lack of investment to find better pharmacologic targets and give them ample opportunity to be studied. People with schizophrenia deserve better, and we as researchers, drug developers, clinicians, and advocates need to work toward getting this important work more resources for treatment development.
